# Factors influencing image quality in fetal cardiovascular magnetic resonance cine imaging using Doppler ultrasound gating: A multicenter study

**DOI:** 10.1016/j.jocmr.2025.101875

**Published:** 2025-03-07

**Authors:** Thomas M. Vollbrecht, Luis F. Goncalves, Dianna M.E. Bardo, Christopher Hart, Heide Boeth, Alex J. Barker, Richard M. Friesen, Julian A. Luetkens

**Affiliations:** a Department of Diagnostic and Interventional Radiology, University Hospital Bonn, Bonn, Germany; b Quantitative Imaging Lab Bonn (QILaB), University Hospital Bonn, Bonn, Germany; c Department of Radiology, Phoenix Children’s Hospital, Phoenix, Arizona, USA; d Department of Medical Imaging, Ann & Robert H. Lurie Children’s Hospital of Chicago, Chicago, Illinois, USA; e Department of Pediatric Cardiology, University Hospital Bonn, Bonn, Germany; f Northh Medical, Hamburg, Germany; g Department of Radiology, Section of Pediatric Radiology, Children’s Hospital Colorado, University of Colorado Anschutz Medical Campus, Aurora, Colorado, USA; h Division of Cardiology, Heart Institute, Children’s Hospital Colorado, University of Colorado School of Medicine, Aurora, Colorado, USA

**Keywords:** Fetal cardiac MRI, Doppler US, Congenital heart disease, Scan protocol

## Abstract

**Background:**

Fetal cine cardiovascularmagnetic resonance (CMR) is an emerging technique for evaluating the fetal heart in conditions such as congenital heart disease, but limited evidence on factors affecting image quality restricts its clinical potential. This study investigated key determinants of image quality in a multicenter cohort.

**Methods:**

This study analyzed fetal CMR scans from April 2021 to July 2023 at three centers (University Hospital Bonn, Children’s Hospital Colorado, Phoenix Children’s Hospital). Cine image quality was assessed using a 5-point Likert scale (1 = non-diagnostic to 5 = excellent) across three criteria as follows: contour sharpness, blood-to-structure contrast, and artifacts. Overall image quality scores were calculated by the average of all criteria. Apparent signal-to-noise (aSNR) and contrast-to-noise ratios (aCNR) were measured. Nine parameters were evaluated for their impact on image quality, namely: gestational age, body mass index (BMI), fetal motion, patient positioning, gating signal stability, breathing technique, field strength, slice thickness, and flip angle. Comparisons were conducted using the Mann-Whitney U test.

**Results:**

A total of 98 scans were analyzed. Higher overall image quality, aSNR, and aCNR were observed in participants with BMI <30 kg/m², gestational age ≥32 weeks, low fetal motion severity, and stable gating signals (e.g., overall image quality for BMI <30 kg/m² vs ≥30 kg/m²: 4.4 ± 0.7 vs. 4.1 ± 0.7, p<0.001). Supine positioning resulted in better overall image quality compared to the left lateral position (4.5 ± 0.5 vs. 4.2 ± 0.8, p = 0.001). Breath-holds provided similar overall image quality but improved contour sharpness and reduced artifacts compared to free breathing (5 [4,5] vs. 4 [4,5], p = 0.042; and 4 [3–5] vs. 4 [3–5], p = 0.014, respectively). At 1.5T field strength, higher contrast and fewer artifacts were observed compared to 3T (5 [4,5] vs. 5 [4,5], p = 0.041; and 4 [4,5] vs. 4 [3–5], p = 0.010, respectively). Slice thickness showed no significant impact on image quality.

**Conclusion:**

Various factors (e.g. BMI) influence fetal cardiac cine MRI image quality. Understanding these factors may help achieving reliable examinations and better exploit the potential of fetal cardiac MRI in clinical routine.

## Graphical abstract

**Figure:**
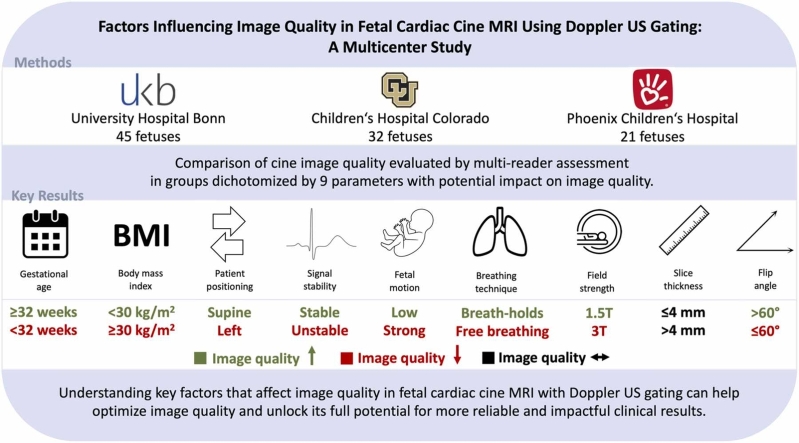

## 1. Introduction

Magnetic resonance imaging (MRI) is an established adjunct to ultrasound for assessing fetal anatomy across various organ systems, which offers excellent soft tissue contrast without known adverse effects on the developing fetus [1], [2]. This is especially relevant when pregnancy is complicated by maternal obesity or in late gestation when decrease in amniotic fluid and ossification of the fetal skeleton impede visualization of fetal structures with ultrasound [3]. Common indications for fetal MRI include the evaluation of brain development and the assessment of thoracic and abdominal abnormalities, such as congenital diaphragmatic hernia or abdominal masses [4], [5], [6]. However, the most common major hereditary anomaly is congenital heart disease (CHD), representing nearly one-third of all such cases, with a reported prevalence ranging from 4 to 10 per 1000 live births [7], [8]. Furthermore, CHD is the leading cause of infant death attributable to birth defects worldwide, with 25% of cases considered critical, requiring surgery or other interventions during the first year of life [9], [10]. Although cardiovascular magnetic resonance (CMR) is the standard of care for morphologic and functional cardiac assessment in both children and adults [11], its application in the fetal population has largely been confined to research settings. In addition to challenges like the small size of fetal structures, a key limitation arises from the need to synchronize CMR data acquisition with the heartbeat to accurately capture cardiac motion, which is difficult in the fetus due to the absence of an MRI-compatible fetal electrocardiogram (ECG). Over the past years, alternative gating methods have been developed as substitutes for ECG gating, including Doppler ultrasound (US) gating [12], [13], [14], [15]. Similar to conventional ECG, this approach enables the adoption of standard CMR sequences and allows for quality review almost in real-time during scanning, as no time-consuming post-processing is required. Consequently, fetal CMR is increasingly being integrated into routine clinical workflows, where it has been shown to detect previously unknown cardiovascular malformations and significantly impact postnatal care [16], [17]. However, inability to sustain a steady gating signal secondary to excessive fetal motion, and artifacts that may originate from fetal motion, maternal breathing motion, pulsations from adjacent maternal vessels, and inherent MRI artifacts can sometimes render scans non-diagnostic. Thus, identifying the optimal conditions to achieve sufficient image quality and minimize artifacts is essential. Nonetheless, specific factors that may influence image quality, such as patient characteristics, CMR scan workflow, and scan parameter choices, have not been systematically investigated. For instance, published studies vary in patient positioning (supine vs. left lateral), breathing technique (free breathing vs. breath-holds), and scanner field strength (1.5 vs. 3T) [18]. The knowledge gap regarding optimal clinical and technical prerequisites limits the potential of fetal CMR and may hinder its widespread implementation in clinical practice. The aim of this study was to investigate the potential influence of examination-related factors on image quality in a large cohort of clinical fetal CMR scans from three centers with differing examination settings.

## 2. Material and methods

### 2.1 Study subjects

This retrospective analysis included fetal CMR scans from April 2021 to July 2023 as a part of a prospective study at three different centers as follows: University Hospital Bonn, Bonn, Germany; Children’s Hospital Colorado, University of Colorado Anschutz Medical Campus, Aurora, Colorado, USA; Phoenix Children’s Hospital, Phoenix, Arizona, USA. All study subjects were referred for clinical fetal CMR following fetal US that raised concern for congenital malformations. The study was approved by the respective institutional review boards at all three sites and all participants provided written informed consent to participate. Exclusion criteria included lack of at least one stack of axial cine images covering the thoracic cardiovascular structures.

### 2.2 Fetal CMR acquisition

All scans were performed using clinical whole-body MRI systems at 1.5 or 3T (University Hospital Bonn: Ingenia 1.5T and Ingenia Elition X 3.0T, Philips Healthcare; Children’s Hospital Colorado: Ingenia 1.5T and Ingenia Elition X 3.0T, Philips Healthcare, Phoenix Children’s Hospital: Achieva dStream 1.5T and Ingenia Elition X 3.0T, Philips Healthcare). Multichannel anterior torso coils and posterior coils integrated into the patient table were used for signal reception. The specific absorption rate was operated in normal mode with a maximum limit of 2.0 W/kg. Depending on patient preference and center experience, participants were examined either in the supine position or the left lateral position. To synchronize CMR data with the fetal cardiac cycle, an external MRI-compatible Doppler ultrasound (DUS) system was employed (smart-sync, Northh Medical GmbH, Hamburg, Germany). The DUS transducer was placed on the maternal abdomen over the presumed location of the fetal heart, either before imaging or following an initial localizer scan to determine the fetal position in utero. Headphones were utilized to audibly monitor the fetal heart during DUS application, facilitating transducer placement over the fetal heart. Once positioned, the DUS transducer was secured firmly with an elastic belt. Subsequently, the anterior coil was positioned with careful consideration to avoid displacement of the DUS transducer during the entire scan. Finally, another localizer scan was performed to confirm the accurate placement of the DUS transducer and for planning purposes.

The imaging protocol employed at all sites included two-dimensional segmented balanced steady-state free precession (bSSFP) cine sequences in an axial orientation perpendicular to the fetal craniocaudal axis, covering the entire heart and major thoracic vessels. In cases where cine stacks were repeated, only those that fully covered the fetal heart and great thoracic vessels were included to ensure comparability between scans. Scan parameters differed among the three sites, depending on prior experience and site preference. In addition to axial cine images, other bSSFP imaging planes and sequences were also obtained for specific study questions but were not part of this study.

### 2.3 Image quality assessment

Multi-reader assessment of image quality was conducted via online video meetings from March to July 2023 using Microsoft Teams software (March 2023 version, Microsoft). In these meetings, 5–10 scans from different centers were shown in random order per weekly session. Care was taken to ensure optimal transfer quality. Via screen sharing, axial cine images of each scan were displayed as a continuous stack directly from the diagnostic image viewer in multiple repetitions to account for potential variations in transfer quality over time. All images were reviewed on diagnostic monitors, and no impairments in spatial or temporal resolution were observed. Before evaluation, each scan was pseudonymized using a study ID. Readers performed their ratings blinded to the ratings of other readers as well as to any clinical or technical scan information. Readers were not permitted to communicate during the rating process. The readers were T.M.V., a cardiovascular radiologist with 4 years of experience in imaging CHD and 3 years in fetal cardiac MRI (reader 1); D.M.E.B, a cardiovascular radiologist with 24 years of experience in imaging CHD and 10 years in fetal CMR (reader 2), R.M.F., a cardiologist with 6 years of experience in imaging CHD and fetal CMR (reader 3), and L.F.G., a pediatric radiologist with 31 years of experience in imaging CHD and 6 years in fetal CMR (reader 4). The levels of image quality throughout the axial cine stack were rated using the following criteria: [1] contour sharpness of cardiovascular structures; [2] blood pool-to-cardiovascular structure contrast; [3] level of artifacts (e.g., blurring, wrap-around artifacts, pulsation artifacts). Details of the scoring points are provided in Table S1. Blinded to qualitative ratings, quantitative image analysis was conducted by reader 1 via a shared server. Apparent signal-to-noise ratio (aSNR) and apparent contrast-to-noise ratio (aCNR) were calculated using the mean signal intensity and standard deviation within equal-sized regions of interest placed in the fetal left ventricular blood pool, fetal interventricular septal myocardium, and maternal abdominal wall or lumbar muscle at end-diastole in the four-chamber view on axial cine images, as previously described [19], [20], [21].

### 2.4 Evaluation of scan information and data analysis

Following image quality assessment, clinical and technical scan information was gathered for each scan at the respective centers. This information was derived from notes documented during scanning, as well as from the pre-examination interview notes and the hospital information system (for maternal and fetal clinical characteristics), and from the digital imaging and communications in medicine (DICOM) header (for scan procedure and parameters). Thus, anonymized data from all centers were collected and analyzed by T.M.V. Based on previously published data, nine examination-related factors were selected to assess their impact on image quality [18]. These items included maternal and fetal characteristics (gestational age, body mass index [BMI], fetal motion), CMR scan procedural conditions (patient positioning, gating signal stability, breathing technique), and CMR imaging parameters (field strength, slice thickness, flip angle). For each comparison, the data were dichotomized into two groups, as needed. Gating signal instability was documented directly during scanning and defined as the presence of short-term signal losses during cine acquisitions, which caused interruptions but did not lead to premature termination of the acquisition. Also, the severity of fetal motion was documented during scanning and categorized on a 5-point scale (1 = none, 2 = minimal, 3 = moderate, 4 = significant, 5 = severe). For analysis purposes, motion severity was dichotomized into low severity (grades 1–2) and high severity (grades 3–5). All comparisons were based on the combined ratings from all readers.

### 2.5 Statistical analysis

Prism (Version 9.5.1; GraphPad Software, Boston, Massachusetts) and SPSS (Version 27, IBM Corp., Armonk, New York) were used for statistical analysis. Dichotomous variables were summarized as percent of absolute frequency. Continuous variables were checked for Gaussian normal distribution using the Shapiro-Wilk test and reported as mean ± standard deviation. Non-parametric discrete variables were reported as median and interquartile range (IQR). Group differences between different centers were assessed using one-way analysis of variance (ANOVA) followed by Tukey multiple comparison tests or Kruskal-Wallis test followed by Dunn’s multiple comparison tests in case of non-parametric data. Image quality scores between dichotomized groups were compared using the Mann-Whitney U test. Inter-reader reproducibility on grades of image quality was assessed using intraclass correlation coefficients (ICC). ICC estimates and their 95% confidence interval (CI) were based on a single-measure, two-way mixed (consistency) model (<0.5: poor, 0.5–0.75: moderate; 0.75–0.9: good, >0.9: excellent). A p-value of <0.05 was considered to indicate a significant difference and a p-value of <0.1 to indicate a tendency.

## 3. Results

**Fig. 1:**
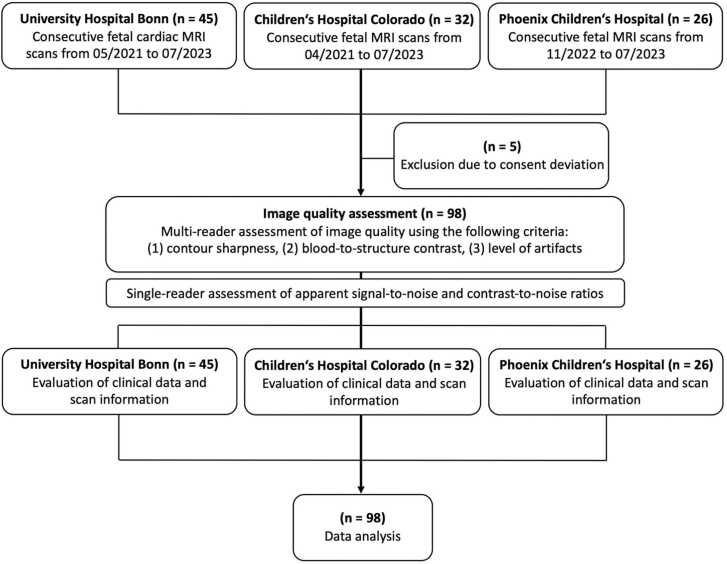
Flowchart of multicenter study participants that were included. *MRI* magnetic resonance imaging

A total of 103 fetal CMR examinations were assessed for image quality, including 45 scans from University Hospital Bonn, 32 scans from Children’s Hospital Colorado, and 26 scans from Phoenix Children’s Hospital. Of these, five scans had to be excluded from further analysis due to consent deviations that could not be resolved by re-consenting (Fig. 1). Thus, the final study collective consisted of 98 subjects with a mean age of 30.7 years ± 5.2. The mean gestational age at the time of the CMR scan was 33.6 weeks ± 3.5. Notably, CMR scans conducted at Phoenix Children’s Hospital were performed significantly earlier in pregnancy compared to those conducted at University Hospital Bonn and Children’s Hospital Colorado (28.6 ± 2.9 weeks vs. 34.9 ± 1.3 weeks vs. 33.7 ± 2.5 weeks, p = <0.001). In 90 of all 98 cases (92%), the reason for referral was to characterize CHD, which was suspected by previous fetal US. In 8 of 98 cases (8%) the reason for referral was to assess cardiac function in congenital diaphragmatic hernia. Maternal and fetal clinical characteristics are given in Table 1.

**Table 1:** Characteristics of study population.

	All subjects	University Hospital Bonn	Children’s Hospital Colorado	Phoenix Children’s Hospital	p-Value
	n = 98	n = 45	n = 32	n = 21	
*Maternal characteristics*					
Age (y)	30.7 ± 5.2	32.5 ± 4.8^a^	30.2 ± 6.0	27.0 ± 3.9^c^	**<0.001**
Weight (kg)	80.6 ± 14.9	80.2 ± 16.3	84.3 ± 13.5	76.2 ± 12.6	0.155
BMI (kg/m^2^)	29.3 ± 6.0	28.9 ± 6.1	30.4 ± 6.9	28.4 ± 3.8	0.415
Gestational age (wk)	33.6 ± 3.5	34.9 ± 1.3^a^	33.7 ± 2.5^a^	28.6 ± 2.9	**<0.001**
*Reason for referral*					
Congenital heart disease	90 (92)	42 (93)	27 (84)	21 (100)	
Congenital diaphragmatic hernia	8 (8)	3 (7)	5 (16)	0 (0)	
*Fetal characteristics*					
Heart rate at CMR (min^−1^)	140 ± 16	138 ± 14	144 ± 9	138 ± 26	0.210
*Sex*					0.675
Female	49 (50)	22 (49)	15 (47)	12 (57)	
Male	49 (50)	23 (51)	17 (53)	9 (43)	
Birth weight (g)	3171 ± 585	3259 ± 608	3133 ± 439	2945 ± 776	0.236
Birth length (cm)	49.7 ± 3.4	50.2 ± 3.4	49.0 ± 2.8	49.5 ± 4.9	0.352
*Mode of delivery*					**0.028**
Vaginal	43 (48)	17 (39)^b^	20 (67)^c^	6 (37)	
Cesarean section	47 (52)	27 (61)	10 (33)	10 (63)	

Data are numbers (%) or mean ± standard deviation. p-values were obtained using one-way analysis of variance followed by Tukey multiple comparison test

*CMR* cardiovascular magnetic resonance*, BMI* body-mass index

a : p = <0.05 to Phoenix Children’s Hospital

b : p = <0.05 to Children’s Hospital Colorado

c : p = <0.05 to University Hospital Bonn

### 3.1 CMR scan procedure

Total scan duration was 62.6 min ± 13.7, with a significant shorter scan time at the University Hospital Bonn compared to the Children’s Hospital Colorado and the Phoenix Children’s Hospital, depending on additional sequences that were not part of this study (55.6 min ± 10.0 vs. 66.1 min ± 11.7 vs. 72.4 min ± 15.9, p = <0.001). 67 of 98 women (68%) were examined in a left lateral position and 31 of 98 women (32%) were in the supine position. Short-term signal losses during cine acquisitions were observed in 56 out of 85 applicable exams (66%), whereas a stable DUS gating signal throughout the entire scan was achieved in 29 out of 85 exams (34%). The median severity of fetal motion was 2 (IQR 1–3). Around 77 of 98 stacks (79%) were performed using breath-holds, while 21 of 98 stacks (21%) were acquired during free breathing. CMR scan information is given in Table 2, and an overview of CMR scan parameters for each center is provided in Table 3.

**Table 2:** Fetal CMR scan information.

	All subjects	University Hospital Bonn	Children’s Hospital Colorado	Phoenix Children’s Hospital	p-Value
	n = 98	n = 45	n = 32	n = 21	
Total scan duration (min)	62.6 ± 13.7	55.6 ± 10.0^a,^^b^	66.1 ± 11.7^c^	72.4 ± 15.9^c^	**<0.001**
*Patient positioning*					**<0.001**
Supine	31 (32)	3 (7)^a^^,^^b^	13 (41)^a^^,^^c^	15 (71)^b^^,^^c^	
Left lateral	67 (68)	42 (93)^a^^,^^b^	19 (59)^a^^,^^c^	6 (29)^b^^,^^c^	
*DUS gating signal stability*					0.087
High	56 (66)	27 (60)	11 (58)	18 (86)	
Low	29 (34)	18 (40)	8 (42)	3 (14)	
Severity of fetal motion (from 1=lowest to 5=highest) *	2 (1–3)	2 (1–3)	2 (1–3)	2 (1–3)	0.322
*Breathing technique*					**<0.001**
Breath-holds	77 (79)	42 (93)^a^	32 (100)^a^	3 (14)^b^^,^^c^	
Free breathing	21 (21)	3 (7)^a^	0 (0)^a^	18 (86)^b^^,^^c^	

Data are numbers (%) or mean ± standard deviation. P-values were obtained using one-way analysis of variance followed by Tukey multiple comparison test

* Data are median and interquartile range. p-values were obtained using

Kruskal-Wallis test followed by Dunn’s multiple comparison test

*DUS* Doppler ultrasonography

a : p = <0.05 to Phoenix Children’s Hospital

b : p = <0.05 to Children’s Hospital Colorado

c : p = <0.05 to University Hospital Bonn

**Table 3:** Fetal CMR cine imaging parameters.

	University Hospital Bonn n = 45	Children’s Hospital Colorado n = 32	Phoenix Children’s Hospital n = 21
Sequence	2D bSSFP cine	2D bSSFP cine	2D bSSFP cine
Field strength (T)	1.5 and 3	1.5 and 3	1.5 and 3
Field of view (mm)	254 × 254	254 × 254	250 × 250
*Pixel spacing (mm)*			
acquired	1.70 × 1.40	1.58 × 1.51	1.80 × 1.70
reconstructed	0.99 × 0.99	0.88 × 0.88	0.99 × 0.99
Slice thickness (mm)	3–5	8	6
Interslice gaps (mm)	0	−4	−2
Slices per stack	9–20	5–14	9–17
Acquisition duration per slice (sec)	7	7	7
Flip angle (°)	45–80	45–75	60
Time of repetition (msec)	4.2	4.6	4.2
Time of echo (msec)	2.1	2.2	2.1
Shot duration (ms)	25.5	36.5	31
Temporal resolution (msec)	17	21	22
Reconstructed cardiac phases	25	20	20
Acceleration method	SENSE	SENSE	SENSE
Acceleration factor	2	2.2	2

*bSSFP* balanced steady-state free precession*, SENSE* sensitivity encoding (vendor-specific parallel imaging technique). Data are given as representative values or ranges.

### 3.2 Evaluation of image quality

**Fig. 2:**
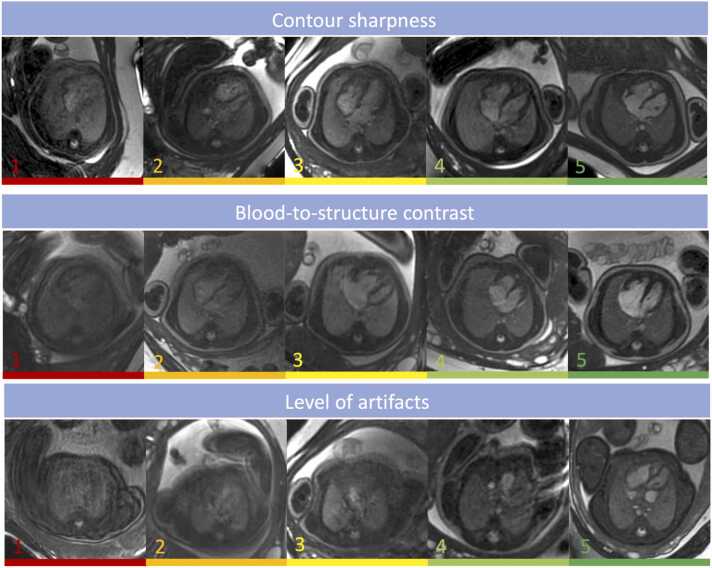
Example images demonstrating the Likert scale gradings from 1 = poor to 5 = excellent for the three categories that were evaluated as follows: [1] contour sharpness, [2] blood pool-to-structure contrast, [3] level of artifacts

All 4 readers provided complete ratings for all 98 included studies in the categories contour sharpness, blood pool-to-structure contrast, and level of artifacts; example images representing all quality grades for the 3 categories are shown in Fig. 2. No significant differences in image quality were observed between centers, as detailed in Table 4.

**Table 4:** Comparison of image quality between centers

	University Hospital Bonn	Children’s Hospital Colorado	Phoenix Children’s Hospital	p-Value
	n = 45	n = 32	n = 21	
*Qualitative metrics*				
Contour sharpness	5 (4–5)	5 (4–5)	4 (4–5)	0.171
Blood-to-structure contrast	5 (4–5)	5 (4–5)	5 (4–5)	0.605
Level of artifacts	4 (3–5)	4 (3–5)	4 (3–5)	0.214
*Quantitative metrics*				
Apparent signal-to-noise ratio	15.5 ± 6.2	16.2 ± 6.8	14.9 ± 7.0	0.778
Apparent contrast-to-noise ratio	27.2 ± 17.1	31.2 ± 17.7	24.9 ± 10.4	0.344

Data are medians with interquartile ranges in parentheses or means ± standard deviation.

Qualitative metrics across all readers are provided. Qualitative metrics were compared by using Kruskal-Wallis tests followed by Dunn’s multiple comparisons test. Quantitative metrics were compared by using analysis of variance (ANOVA) followed by Tukey’s multiple comparison test

### 3.3 Multi-factorial comparison of cine image quality between fetal CMR scans

Significant differences were observed in all comparisons regarding both maternal and fetal conditions. Specifically, overall image quality was significantly higher in subjects with a BMI <30 kg/m^2^ compared to those with a BMI ≥30 kg/m^2^ (4.4 ± 0.7 vs 4.1 ± 0.7, p = <0.001). Additionally, overall image quality was significantly higher in studies involving fetuses with a gestational age ≥32 weeks compared to those with gestational age <32 weeks (4.3 ± 0.7 vs 4.1 ± 0.8, p = <0.001). Finally, image quality was significantly better in exams with low severity of fetal motion compared to exams with severe fetal motion (4.5 ± 0.6 vs 3.9 ± 0.8, p = <0.001).

Regarding factors of the CMR scan procedure, overall image quality was significantly higher in examinations where the women were in supine position compared to those in left lateral position (4.5 ± 0.5 vs 4.2 ± 0.8, p = 0.001). The overall image quality was also better in studies with a stable DUS gating signal compared to those with an unstable DUS gating signal (4.5 ± 0.6 vs 4.0 ± 0.8, p = <0.001). No significant difference in overall image quality was observed between the two breathing techniques (4.3 ± 0.7 vs 4.1 ± 0.7 p = 0.055). However, images acquired during breath-holds had significantly sharper contours and fewer artifacts compared to those obtained during free breathing (5 [4], [5] vs 4 [4], [5], p = 0.042 and 4 [3], [4], [5] vs 4 [3], [4], [5], p = 0.014, respectively).

Similarly, no significant difference was observed regarding field strength (4.3 ± 0.7 vs 4.2 ± 0.7, p = 0.088), although scans at 1.5T showed significantly higher contrast and fewer artifacts compared to 3T field strength (5 [4], [5] vs 5 [4], [5], p = 0.041 and 4 [4], [5] vs 4 [3], [4], [5], p = 0.010, respectively). No significant differences at all were found between cine stacks with ≤4 mm slice thickness compared to cine stacks >4 mm slice thickness (e.g., overall image quality: 4.3 ± 0.7 vs 4.3 ± 0.7, p = 0.840). In contrast, overall image quality was significantly higher when a flip angle >60° was used compared to acquisitions with a flip angle ≤60° (4.4 ± 0.6 vs 4.2 ± 0.8, p = 0.048).

**Fig. 3:**
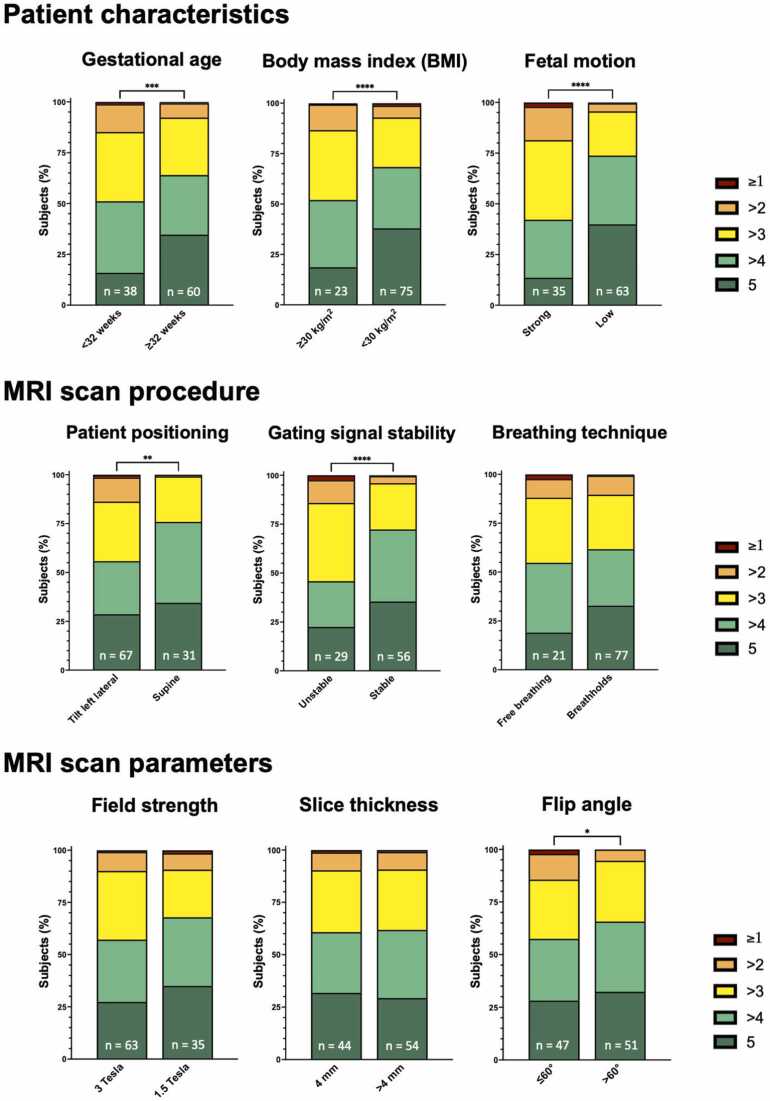
Stacked bar charts show the distribution of Likert scale scores used for overall image quality assessment. Likert scale scores for overall image quality were averaged between the individual ratings for the three categories contour sharpness, blood pool-to-structure contrast, and level of artifacts from all readers. P-values were calculated using the Wilcoxon test. *BMI* body mass index, *MRI* magnetic resonance imaging

**Fig. 4:**
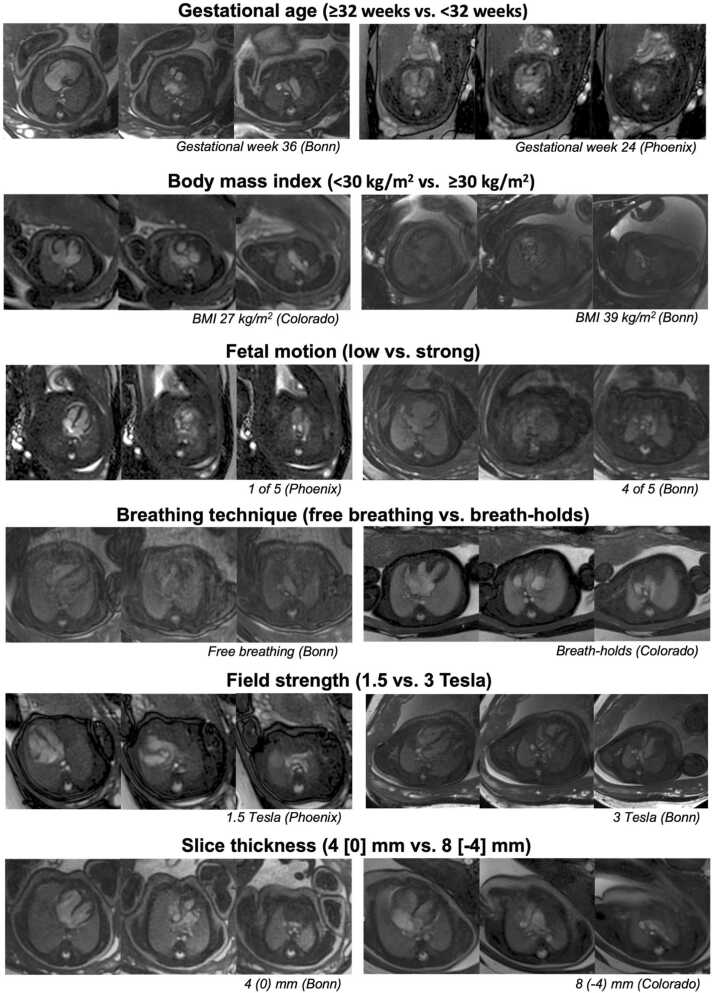
Balanced steady-state free precession cine image examples from the three centers University Hospital Bonn (Bonn), Children’s Hospital Colorado (Colorado), and Phoenix Children’s Hospital (Phoenix), each in one slice at ventricular level, outflow tract level, and aortic arch or ductus arteriosus level. The images from gestational weeks 36 and 24 show the clear improvement of structure delineability as pregnancy progresses. Images of participants with different body mass index demonstrate the influence of obesity on image quality resulting from poorer MR signal. Strong fetal may render images non-diagnostic due to low contrast and pronounced artifacts, as seen here particularly in the slice at the outflow tract level. Images acquired during free breathing are inferior to those acquired during breath-holds because they are affected by poor contour sharpness and linear artifacts caused by maternal abdominal movements associated with breathing. Images acquired at 1.5T had higher contrast and fewer artifacts; however, the overall image quality was not significantly different. When comparing the images by slice thickness, no differences were found in any of the three categories

Bar charts showing the distribution of overall image quality scores for each factor are displayed in Fig. 3. Representative cine images showing quality differences between factors are presented in Fig. 4. Detailed results for all image quality categories are provided in Table 5. Results for aSNR and aCNR are provided in Table 6.

**Table 5:** Multi-factorial analysis of qualitative image evaluation ratings by combined readers.

Factors	Image quality score		p-Value
Overall	4.3 ± 0.7	4.1 ± 0.8	**<0.001**
Contour sharpness	5 (4–5)	4 (3.25–5)	**<0.001**
Contrast	5 (4–5)	4 (4–5)	**0.025**
Artifacts	4 (3–5)	4 (3–5)	**0.006**
Body mass index	<30 kg/m^**2**^	≥30 kg/m^**2**^	**p-Value**
Overall	4.4 ± 0.7	4.1 ± 0.7	**<0.001**
Contour sharpness	5 (4–5)	4 (4–5)	**<0.001**
Contrast	5 (4–5)	4 (4–5)	**0.001**
Artifacts	4 (4–5)	4 (3–5)	**0.001**
Patient positioning	Supine	Left lateral	**p-Value**
Overall	4.5 ± 0.5	4.2 ± 0.8	**0.001**
Contour sharpness	5 (4–5)	4 (4–5)	**0.020**
Contrast	5 (4–5)	5 (4–5)	**<0.001**
Artifacts	4 (4–5)	4 (3–5)	**<0.001**
Doppler US gating signal stability	Stable	Unstable	**p-Value**
Overall	4.5 ± 0.6	4.0 ± 0.8	**<0.001**
Contour sharpness	5 (4–5)	4 (4–5)	**<0.001**
Contrast	5 (4–5)	4 (4–5)	**<0.001**
Artifacts	4 (4–5)	4 (3–4.75)	**<0.001**
Fetal motion	Low	Strong	**p-Value**
Overall	4.5 ± 0.6	3.9 ± 0.8	**<0.001**
Contour sharpness	5 (4–5)	4 (3–5)	**<0.001**
Contrast	5 (4–5)	4 (4–5)	**<0.001**
Artifacts	4 (4–5)	4 (3–4)	**<0.001**
Breathing technique	Breath-holds	Free breathing	**p-Value**
Overall	4.3 ± 0.7	4.1 ± 0.7	0.055
Contour sharpness	5 (4–5)	4 (4–5)	**0.042**
Contrast	5 (4–5)	4 (4–5)	0.142
Artifacts	4 (3–5)	4 (3–5)	**0.014**
Field strength	1.5 Tesla	3 Tesla	**p-Value**
Overall	4.3 ± 0.7	4.2 ± 0.7	0.088
Contour sharpness	4.5 (4–5)	4 (4–5)	0.913
Contrast	5 (4–5)	5 (4–5)	**0.041**
Artifacts	4 (4–5)	4 (3–5)	**0.010**
Slice thickness	≤4 mm	>4 mm	**p-Value**
Overall	4.3 ± 0.7	4.3 ± 0.7	0.840
Contour sharpness	5 (4–5)	4 (4–5)	0.268
Contrast	5 (4–5)	5 (4–5)	0.431
Artifacts	4 (3–5)	4 (3–5)	0.935
Flip angle	>60°	≤60°	**p-Value**
Overall	4.4 ± 0.6	4.2 ± 0.8	**0.048**
Contour sharpness	5 (4–5)	4 (4–5)	**0.001**
Contrast	5 (4–5)	5 (4–5)	0.216
Artifacts	4 (4–5)	4 (3–5)	0.176

Overall image quality scores, averaged from equally weighted qualitative metrics, are shown as means ± standard deviation. Qualitative metrics are reported as medians with interquartile ranges. Qualitative metrics across all readers are provided. Mann-Whitney U test was used for comparisons. Bold indicates statistical significance (p = <0.05)

*US* ultrasonography

**Table 6:** Multi-factorial comparison of quantitative image quality metrics.

Factors	Image quality score		p-Value
Apparent signal-to-noise ratio	16.0±6.3	12.9±4.3	**0.034**
Apparent contrast-to-noise ratio	29.3±16.9	21.3±8.5	**0.034**
Body mass index	<30 kg/m^**2**^	≥30 kg/m^**2**^	**p-Value**
Apparent signal-to-noise ratio	16.6±6.8	13.9±5.5	**0.041**
Apparent contrast-to-noise ratio	30.4±17.0	23.5±12.6	**0.035**
Patient positioning	Supine	Left lateral	**p-Value**
Apparent signal-to-noise ratio	15.5±3.9	15.6±7.3	0.969
Apparent contrast-to-noise ratio	28.5±9.8	27.8±18.2	0.845
Doppler US gating signal stability	Stable	Unstable	**p-Value**
Apparent signal-to-noise ratio	17.4±6.7	12.9±4.7	**0.002**
Apparent contrast-to-noise ratio	32.7±15.1	19.8±11.6	**<0.001**
Fetal motion	Low	Strong	**p-Value**
Apparent signal-to-noise ratio	16.9±6.5	13.2±5.6	**0.006**
Apparent contrast-to-noise ratio	31.2±15.8	22.3±14.2	**0.006**
Breathing technique	Breath-holds	Free breathing	**p-Value**
Apparent signal-to-noise ratio	15.5±6.5	15.0±7.0	0.765
Apparent contrast-to-noise ratio	28.3±17.0	24.1±10.8	0.281
Field strength	1.5 Tesla	3 Tesla	**p-Value**
Apparent signal-to-noise ratio	15.6±7.0	15.2±6.2	0.738
Apparent contrast-to-noise ratio	28.1±15.1	26.6±16.3	0.650
Slice thickness	≤4 mm	>4 mm	**p-Value**
Apparent signal-to-noise ratio	15.4±6.2	15.7±6.7	0.800
Apparent contrast-to-noise ratio	27.0±17.2	28.3±14.6	0.696
Flip angle	>60°	≤60°	**p-Value**
Apparent signal-to-noise ratio	16.1±6.5	14.8±6.5	0.331
Apparent contrast-to-noise ratio	30.9±16.6	24.1±14.1	**0.034**

Quantitative metrics are mean ± standard deviation. Bold indicates statistical significance (p = <0.05)

*US* ultrasonography

### 3.4 Inter-reader reproducibility

Reproducibility among all readers was good for the image quality ratings in all categories. ICC results are presented in Table 7.

**Table 7:** Inter-reader reproducibility for the qualitative image evaluation ratings of the different readers.

	Intraclass correlation coefficient	95% confidence interval	
		Lower bound	Upper bound
Contour sharpness	0.816	0.751	0.868
Contrast	0.772	0.690	0.836
Artifacts	0.869	0.823	0.906

Data representing reproducibility assessments based on intraclass correlation coefficients with corresponding 95% confidence intervals

## 4. Discussion

Fetal CMR is an emerging technique for characterizing cardiac malformations in prenatal life. Considering the complex anatomy often involved, high-quality cine imaging is an essential constituent for precise diagnostic assessment. However, there is limited knowledge about best practices for achieving optimal image quality, given the varying examination settings observed in earlier studies. This study identified various factors with potential influence on image quality in DUS-gated fetal cine CMR, including degree of fetal motion, gestational age, maternal weight, DUS gating signal stability, patient positioning, breathing technique, field strength, and choice of flip angles.

The development of gating strategies replacing traditional ECG has facilitated the use of dynamic fetal CMR in both research and clinical practice [16], [22], [23], [24], [25], [26], [27], [28], [29]. However, studies have documented a significant number of examinations that were prematurely terminated due to unpredictable fetal motion [30], [31]. This issue is also evident in our study, where high levels of fetal movement during the examination were associated with significant degradation of image quality. Unlike other factors, fetal motion remains a challenging aspect that is difficult for the examiner to control. Troubleshooting strategies include waiting for quieter fetal periods, repetition of the DUS transducer placement, or changing the phase-encoding direction to project the artifacts to less critical areas of the image [18]. Additionally, in the current software version, missing signals can be automatically substituted by artificial trigger injection; however, this feature was not yet available at the time of this study. Moreover, technical options such as non-Cartesian acquisition schemes and motion compensation techniques might be applied in the future, but are currently limited by computational cost and time-demanding post-processing [23].

A gestational age of less than 32 weeks was identified as another factor related to impaired image quality, particularly regarding contour sharpness. This finding aligns with previous studies where the gestational age of the fetuses typically ranged after week 31 [17], [18], [32], [33], [34]. The reasons for this are likely due to the decreasing amount of amniotic fluid and the increasing size of fetal cardiovascular structures. Conversely, fetal echocardiography often faces limitations in advanced pregnancy due to increasing bone calcification and adverse fetal positions, which affect acoustic penetration and window quality. Therefore, restricting CMR to the third trimester can be considered a valuable addition to prenatal care before birth; e.g., it allows for the assessment of heart defects that may not be apparent in the second trimester, such as valve stenosis, or for evaluating the effects of changing fetal hemodynamics in late pregnancy [35], [36].

Increased maternal BMI >30 kg/m² was identified as another factor linked with significantly poorer image quality. This is likely due to the longer distance of the reception coil and DUS transducer from the fetal heart, as well as potentially reduced breathing capacity. Obesity has become the most prevalent clinical risk factor in obstetric practice, associated with complications in late pregnancy and adverse outcomes for both mother and newborn. Therefore, imaging plays a crucial role in the clinical management of obese pregnant women [37]. Consequently, users of fetal CMR will frequently encounter this challenge in practice, especially considering the strong association between maternal obesity and CHD. In these cases, optimal positioning is critical to minimize the distance between the coil and DUS transducer and the fetal heart, and to adjust the field of view to prevent aliasing.

Another critical factor potentially influencing image quality is the stability of the DUS signal. Unlike ECG, the DUS method is highly dependent on the position of the fetal heart, which changes due to varying respiratory depth and fetal movement, resulting in frequent signal instabilities. In most cases, the DUS device quickly regains the signal, resulting in only brief interruptions without causing termination of data acquisition. However, these short signal losses lasting about 3 s increase the acquisition time per slice, as observed in this study where it extended up to 22 s. The prolonged acquisition time may affect image quality due to the influence of respiration and fetal movement. To address these short signal losses, current software versions mitigate the issue by allowing for adjustable durations of injected triggers based on the previous heart rate. Other potential solutions include increasing the size of the DUS transducer to create a larger acoustic beam and enabling depth selection for a stronger focus on the fetal heart, which may be incorporated in future software updates. Alternative gating methods, such as metric-optimized and self-gating strategies, can be employed but are also susceptible to fetal movements and typically do not allow immediate online image review due to the need for retrospective trigger point extraction [14], [38].

Longer signal losses can abruptly terminate data acquisition, necessitating sequence repetition and potentially requiring correction of the DUS transducer position, as observed in 5 cases in this study.

Therefore, it is crucial to position the DUS transducer as precisely as possible centered above the fetal heart before starting the scan and to verify its position using localizers in cases of repeated signal losses.

As in most other studies, participants were scanned either in supine position or in left lateral position, according to patient preference and center experience. Interestingly, examinations in the supine position showed significantly higher image quality, especially in terms of contrast and artifacts, but showed no difference in quantitative image quality metrics. This can be attributed to better maternal stability, which reduces motion artifacts, increased comfort during the examination, and less influence of breathing on body movement, rather than technical factors such as improved anterior torso coil coverage or more even distribution of body mass within the magnetic field. However, it is important to consider the woman's preference in individual cases, as the potential advantages of the supine position may be outweighed by reduced comfort. Balancing these factors is essential to optimize image quality while ensuring patient comfort and cooperation during the examination.

Another factor that impacts patient comfort is the breathing strategy used during cine acquisitions. In this study, the use of breath-holds tended to result in better overall image quality, with significantly higher contrast and reduced artifact burden. However, the lower levels of significance and comparable quantitative metrics observed with both breathing strategies suggest that fetal movement, as another major source of artifacts, exerts a greater influence on image quality. This may be because, despite using breath-hold maneuvers, pregnancy often leads to insufficient breath suppression, as evidenced by previous findings where breathing artifacts were present in up to 22% of examinations despite attempts at breath-holding [16]. While the results suggest that a breath-hold technique should generally be used if well tolerated by the pregnant woman, the additional impairments associated with free breathing appear to be acceptable if appropriate adjustments, such as increasing the signal averages, are made. Additionally, complex image reconstruction pipelines involving free breathing and motion compensation are feasible, but lack of widespread availability and long reconstruction times currently limit their clinical value [32].

Both 1.5T and 3T MRI systems were used for fetal CMR in this study, as well as in previous studies [12], [17], [27], [30], [34], [35], [39]. In theory, the benefits of higher field strength particularly apply to fetal imaging, considering the small sizes of structures, high heart rates, and eventual complex anatomy. These include higher spatial and temporal resolution, shorter acquisition time, and a higher contrast-to-noise ratio based on longer T1 relaxation times [40]. However, both aSNR and aCNR were comparable between the two field strengths, with no advantage at 3T MRI. Moreover, there was a tendency towards better subjective image quality at 1.5T compared to 3T, with significant differences observed in contrast and artifact reduction. This suggests that challenges associated with higher field strengths, such as pronounced susceptibility and dielectric shielding effects due to increased B0 and B1 inhomogeneities, are more prominent with cine bSSFP in the fetus. Additionally, factors like dielectric shielding may be exacerbated by common concurrent conditions in pregnancy, such as polyhydramnios. Whether theoretical advantages of 3T may outweigh the disadvantages in other sequences, such as in fetal phase-contrast flow imaging, should be investigated in the future.

Consistent with previous studies, a wide range of flip angles were used in this study, with flip angles >60° significantly improving contour sharpness and aCNR [34], [39]. Although subjective contrast was comparable between the two groups, the enhanced edge definition between blood and cardiovascular structures likely result from increased contrast between myocardial and blood signal intensities, providing a simple yet effective way to influence image quality. However, this must be balanced against potential drawbacks, such as increased specific absorption rate due to elevated radiofrequency energy deposition, longer scan times, or reduced temporal resolution due to prolonged repetition times. In contrast, no differences in image quality were found with different slice thickness regimens. This could be because the theoretical advantage of higher SNR with slice thicknesses of 6 and 8 mm compared to ≤4 mm is offset by increased partial volume effects. Conversely, whether the higher spatial resolution of ≤4 mm slice thickness without gaps actually leads to a better assessment of subtle cardiovascular structures such as valves or pulmonary vessels must be investigated in further studies.

## 5. Limitations

Despite predefined image quality grades and multiple test ratings, a limitation of this study lies in the inherent subjectivity of qualitative assessments, which could have introduced bias from readers' expectations and prior experiences with certain imaging techniques. To mitigate this, readers were blinded to scan information, and a combined reader approach with four experienced readers from different centers was employed. Additionally, dichotomizing scans based on investigated factors may have resulted in the loss of information and statistical power for continuous variables like gestational age and BMI, where clinical cut-offs were applied. Potential interdependencies between factors, such as fetal motion and gating signal stability, were not explicitly accounted for, although robust associations across a large multicenter dataset suggest independent effects on image quality. The use of MRI systems from a single vendor across all sites may limit generalizability, but reduces potential confounders related to variations in techniques and hardware performance. Thus, the identified factors are likely applicable to scans using MRI systems from other vendors, and future studies should validate these findings with other MRI systems. Lastly, other imaging techniques of fetal cardiac MRI, such as phase-contrast imaging or slice-to-volume reconstructions, were not included in the study and may have influenced the results.

## 6. Conclusion

In conclusion, this study fills a gap in the knowledge required for optimizing image quality in the clinical use of DUS-gated fetal CMR. The findings suggest that optimal image quality can be achieved in fetuses older than 32 weeks gestation, examined in the supine position of a normal-weight mother, during breath-holds at 1.5T, using a flip angle of 60° or more. Even if some of these conditions are not met, the examiner should be aware of them and able to make adjustments to maximize the potential of fetal cardiac MRI, even under challenging examination conditions.

## Funding

None.

## Author contributions

**Alex J. Barker:** Writing – review & editing, Resources, Investigation, Data curation, Conceptualization. **Richard M. Friesen:** Writing – review & editing, Resources, Investigation, Data curation, Conceptualization. **Julian A. Luetkens:** Writing – review & editing, Validation, Supervision, Project administration, Methodology, Conceptualization. **Luis F. Goncalves:** Writing – review & editing, Resources, Investigation, Data curation, Conceptualization. **Thomas M. Vollbrecht:** Writing – original draft, Visualization, Validation, Resources, Project administration, Methodology, Investigation, Formal analysis, Data curation, Conceptualization. **Dianna M. E. Bardo:** Writing – review & editing, Resources, Investigation, Conceptualization. **Christopher Hart:** Writing – review & editing, Conceptualization. **Heide Boeth:** Writing – review & editing.

## Declaration of competing interests

The authors declare the following financial interests/personal relationships which may be considered as potential competing interests: H.B. is employed by Northh Medical, the company that markets the Doppler US device. H.B. was involved in the study organization, but had no access to the clinical or technical study data, nor any influence on the data analysis or interpretation.
