# Supplementary material for: Factors influencing image quality in fetal cardiovascular magnetic resonance cine imaging using Doppler ultrasound gating: A multicenter study
Source: J Cardiovasc Magn Reson. 2025 Mar 7;27(1):101875. doi: 10.1016/j.jocmr.2025.101875 (PMC12053712; doi:10.1016/j.jocmr.2025.101875)
Supplement: Supplementary file 1 — Supplementary material [file mmc1.docx]

# Supplementary material

**Table S1.** Likert scale scoring points used for image quality assessments

| **Score** | **Blood pool-to-cardiovascular structure contrast** | **Contour sharpness of cardiovascular structures** | **Level of artifacts** |
| --- | --- | --- | --- |
| 5 (Excellent) | Blood pool is hyperintense with excellent contrast against the cardiovascular structures in all images. | Contours of cardiovascular structures are sharply defined in all images, no blurring. | Stack is almost artifact free. |
| 4 (Good) | Blood pool is significantly brighter than the cardiovascular structures wall in most images. | Contours of cardiovascular structures are discernible in most images, minimal blurring. | Few artifacts are present that do not affect delineation cardiovascular structures |
| 3 (Moderate) | Blood-pool-to-structure contrast features significant variations in some images, but stack is of diagnostic quality | Contours of cardiovascular structures are barely distinguishable in few images, but stack is of diagnostic quality | Artifacts are present that hinder delineation of cardiovascular structures in some images. |
| 2 (Poor) | Blood pool is barely distinguishable from cardiovascular structures in most images, severely affecting image. | Cardiovascular contour sharpness is poor in most images, severely affecting image quality. | Artifacts are present that hinder delineation of cardiovascular structures in most images. |
| 1 (Non-diagnostic) | Blood pool-to-structure contrast is poor; stack is of non-diagnostic quality. | Cardiovascular contour sharpness is poor; stack is of non-diagnostic quality. | Artifacts are present that render the stack non-diagnostic. |
